# Surgical Treatment of Double-Layered Lateral Meniscus

**DOI:** 10.1155/2021/9978889

**Published:** 2021-10-22

**Authors:** Masataka Ota, Hiroshi Takagi, Shin Kato, Fumiyoshi Kawashima, Koji Kanzaki

**Affiliations:** Department of Orthopedic Surgery, Showa University Fujigaoka Hospital, Japan

## Abstract

This is a rare case of a patient with a double-layered lateral meniscus, undergoing surgical treatment. A 17-year-old woman who was a member of a volleyball club had a two-year history of right knee pain with episodes of locking, although she had no history of trauma. She was referred to our hospital because her condition did not improve after conservative treatment. On presentation to the hospital, she had full range of motion in the right knee. McMurray's test revealed no clicks; however, it produced pain in the lateral part. Plain radiography revealed no abnormal findings, whereas magnetic resonance imaging showed high signal in the posterior segment of the lateral meniscus and an increase in its volume. Arthroscopic findings showed an accessory meniscus with a flat surface overlying a normal-sized lateral meniscus. It was firmly connected to the posterior root and middle segment of the lower normal meniscus. The accessory meniscus was markedly mobile as revealed by probing. The patient was diagnosed with double-layered lateral meniscus and underwent resection of the accessory meniscus. Postoperatively, she initiated strengthening of muscles and range of motion training without weight-bearing restrictions. Two months postoperatively, she had completely recovered and participated in volleyball practices. In the last follow-up at 18 months, she had no restrictions in daily or sports activities.

## 1. Introduction

Several types of abnormally shaped meniscus have been reported, mainly in the Asian population, and these more frequently develop in the lateral meniscus than in the medial meniscus [1]. Discoid meniscus is the most common meniscus abnormality, with an incidence of 0.4%–17%. Other abnormally shaped meniscus include ring-shaped and accessory meniscus. The double-layered meniscus is one of these abnormalities, with an incidence of ≤0.1% [2]. Here, we presented the case of a patient who underwent arthroscopic partial meniscectomy for a double-layered lateral meniscus along with a review of the relevant literature.

## 2. Case Report

**Figure 1:**
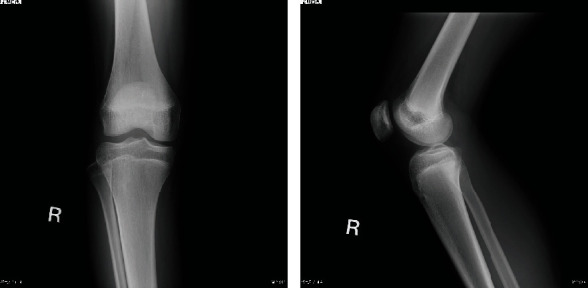
Plain radiography reveals no abnormal findings.

**Figure 2:**
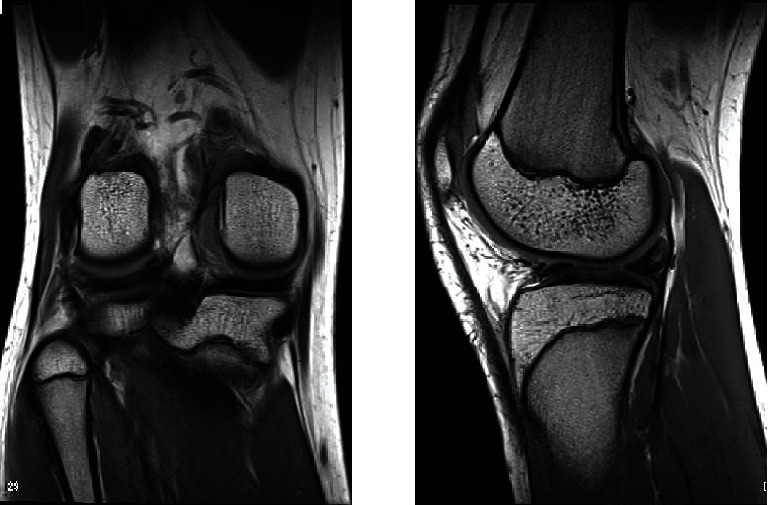
Magnetic resonance imaging (proton density-weighted image) shows high signal in the posterior segment and an increase in its volume.

**Figure 3:**
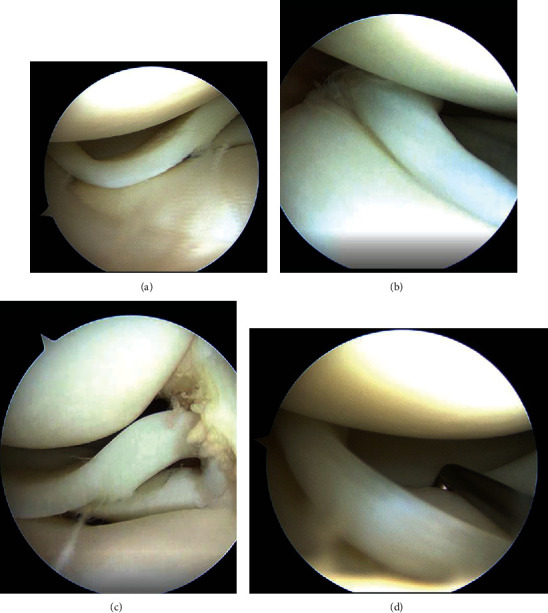
(a) Arthroscopic findings show an accessory meniscus with a flat surface overlying a normal lateral meniscus. (b) and (c) The accessory meniscus is firmly connected to the posterior horn and middle segment of the normal meniscus. (d). The accessory meniscus demonstrated favorable mobility by probing.

**Figure 4:**
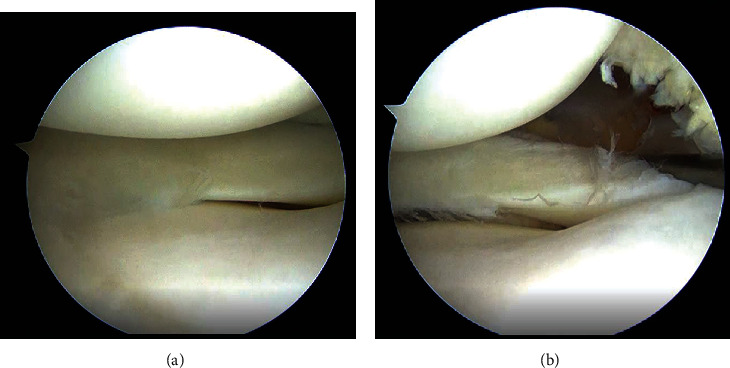
(a) and (b) Arthroscopic findings after resection of the accessory meniscus show that thickness and shape of the resected meniscus are similar to those of the normal meniscus.

A 17-year-old woman, who was a member of a volleyball club, presented with right knee pain. She had no history of trauma. She developed right knee pain with episodes of locking during volleyball practices for 2 years. She was referred to our hospital after conservative treatment in a local clinic. She had no past medical history. Physical findings at initial examination, no swelling or patellar ballottement was observed in the right knee joint, with extension and flexion of 0° and 155°, respectively. There was tenderness in the lateral tibiofemoral joint. McMurray's test revealed no clicks but produced pain in the lateral side. Lachman test was negative, and varus and valgus stress test did not show instability. Plain radiography revealed no apparent abnormal findings (Figure 1). Magnetic resonance imaging (MRI) (proton density-weighted image) showed high signal in the posterior segment of the lateral meniscus and an increase in its volume (Figure 2). Based on these findings, we suspected discoid meniscus injury in the right knee joint, and arthroscopic surgery was performed. In the arthroscopic findings, no abnormal findings were observed in the suprapatellar bursa, patellofemoral joint, medial compartment, or intercondylar fossa. Furthermore, the anterior and posterior cruciate ligaments were normal. In the lateral compartment, an accessory meniscus with a flat surface overlying a normal-sized lateral meniscus extended from the middle segment to the posterior horn. The thickness of these two menisci was similar, and the accessory meniscus was firmly connected to the posterior horn and middle segment of the lower normal meniscus. Furthermore, it showed favorable mobility on probing (Figures 3(a)–3(d)). We diagnosed the double-layered lateral meniscus, and only the accessory meniscus was resected. The remaining meniscus was stable, and its thickness and shape were similar to those of the normal meniscus (Figures 4 (a) and 4 (b)). Strengthening of muscles and range of motion training without weight-bearing restrictions were initiated on the day after surgery. The patient did not complain of pain and had completely recovered and participated in volleyball practices two months after surgery. At three months after surgery, McMurray's test revealed no clicks or pain, and the Lysholm score was 100. In the last follow-up at 18 months after surgery, the patient had no restrictions in daily or sports activities.

## 3. Discussion

The lateral discoid meniscus is the most common meniscus abnormality that commonly affects the Asian population (accounting for 38% of all abnormally shaped menisci) [3]. In 1974, Baily and Blundell initially described the double-layered meniscus as an accessory meniscus [4]. The incidence of the double-layered lateral meniscus is extremely rare, at ≤1%. To the best of our knowledge, only 18 knees in 15 patients have been described in English literature. Some of these were reported in Asian populations. As with discoid meniscus, double-layered lateral meniscus more frequently occurs in men than in women. Of the aforementioned 15 patients, 12 are men. Regarding the onset age, 60% of such cases occur in patients aged ≤20 years, and the youngest patient was aged 8 years [5, 6]. Double-layered meniscus comprises connected and separated morphological types [7]. In the former type, the upper accessory meniscus is connected to the anterior and posterior edges of a lower normal meniscus and not connected to the surrounding area. In contrast, the accessory and normal menisci are not connected to each other in the latter type. The incidence of the connected type is approximately double that of the separated type. Previous studies reported that patients with the connected-type double-layered meniscus develop an accessory meniscus in the lower layer of the normal meniscus [6] and a ring-shaped accessory meniscus [8]. In the present case, proton density-weighted MRI revealed high signal in the posterior segment of the lateral meniscus and an increase in its volume, suggesting the tear of discoid lateral meniscus. According to a previous reports [5], MRI findings of the double-layered meniscus are similar to those with horizontal or bucket-handle tears of the meniscus and normal meniscus. From our experience and these reports, it may be difficult to make a diagnosis of double-layered meniscus based on MRI findings alone. The upper accessory meniscus is a fibrocartilaginous structure composed of dense collagen fibers without derangement in fibers. Thus, its histological findings are consistent with those of meniscus tissue [2]. Symptoms of double-layered meniscus remarkably improve after resection of the accessory meniscus. In our patient, the accessory meniscus was classified as the connected type, and it was firmly connected only with the posterior horn and middle segment of the normal meniscus. Given such a condition, the abnormal mobility of the accessory meniscus may have caused pain and locking. The patient had completely recovered and participated in sports practices after undergoing resection of the accessory meniscus. A previous study had reported mid- and long-term results (mean postoperative period of 10.1 years) of meniscectomy with symptomatic discoid lateral meniscus repair [9]. The results showed that clinical symptoms improve, although osteoarthritis develops in 23% of cases. Previous reports on patients with double-layered meniscus [7, 8] revealed that plain radiography performed approximately one year after surgery did not show any signs of osteoarthritis. However, no mid- and long-term reports on these patients are available. Our patient had no restrictions in performing daily or sports activities at 18 months after surgery. However, long-term follow-up will be required.

## 4. Conclusion

We present the case of a patient who underwent arthroscopic partial meniscectomy for a double-layered lateral meniscus, which is an abnormally shaped meniscus with extremely low incidence. The patient had no restrictions in performing daily or sports activities after resection of accessory meniscus.
